# Periodontal disease and obstructive sleep apnea: an umbrella review

**DOI:** 10.3389/froh.2026.1780859

**Published:** 2026-03-26

**Authors:** Franz Tito Coronel-Zubiate, Carlos Alberto Farje-Gallardo, Paul Martín Herrera-Plasencia, Teresa Etelvina Ríos-Caro, Sonia Tejada-Muñoz, Betsy Quispe-Quispe, Rubén Aguirre-Ipenza, Heber Isac Arbildo-Vega

**Affiliations:** 1 Faculty of Health Sciences, Stomatology School, Universidad Nacional Toribio Rodríguez de Mendoza de Amazonas, Chachapoyas, Peru; 2 Faculty of Health Science, Stomatology School, Universidad César Vallejo, Piura, Peru; 3 Faculty of Stomatology, Stomatology School, Universidad Nacional de Trujillo, Trujillo, Peru; 4 Faculty of Health Sciences, Nursing School, Universidad Nacional Toribio Rodríguez de Mendoza de Amazonas, Chachapoyas, Peru; 5 Department of General Dentistry, Dentistry School, Universidad Nacional del Altiplano, Puno, Peru; 6 Faculty of Health Sciences, Universidad Continental, Lima, Peru; 7 Faculty of Dentistry, Dentistry School, Universidad San Martín de Porres, Chiclayo, Peru; 8 Faculty of Human Medicine, Human Medicine School, Universidad San Martín de Porres, Chiclayo, Peru; 9 Graduate School, Universidad Nacional Toribio Rodríguez de Mendoza de Amazonas, Chachapoyas, Peru

**Keywords:** obstructive sleep apnea, OSA, periodontal disease, periodontitis, review

## Abstract

**Aim:**

To synthesize and critically appraise the existing evidence on the association between periodontal disease (PD) and obstructive sleep apnea (OSA) through an umbrella review.

**Material and methods:**

A comprehensive search was conducted across databases including PubMed, Cochrane Library, Scopus, Web of Science, Embase, SciELO, Google Scholar, ProQuest Dissertations and Theses, and OpenAIRE, covering literature up to December 2025. Systematic reviews, with or without meta-analysis, that assessed the association between PD and OSA were included. Narrative reviews, rapid reviews, clinical trials, observational or experimental studies, case reports, editorials, letters, protocols, and posters were excluded. The methodological quality of the reviews was evaluated using the AMSTAR-2 tool, and the risk of bias was assessed using the ROBIS tool.

**Results:**

From an initial retrieval of 217 records, 11 systematic reviews met the inclusion criteria. The included reviews consistently reported a positive association between PD and OSA, although variability in diagnostic criteria and study design was observed.

**Conclusion:**

Based on systematic reviews with low risk of bias and high overall confidence, the available evidence indicates a consistent association between PD and OSA. However, due to the predominantly observational nature of the primary studies, causality cannot be inferred, and further longitudinal and interventional research is warranted.

**Systematic Review Registration:**

Open Science Framework (OSF), DOI: 10.17605/OSF.IO/RMQWC, URL: https://osf.io/udyjq/.

## 1 Introduction

Periodontal disease (PD) and obstructive sleep apnea (OSA) represent two chronic conditions with a substantial global health burden, whose coexistence in clinical populations is increasingly frequent (1, 2). While PD affects approximately 20%–50% of the global population as one of the most prevalent inflammatory pathologies (3), OSA impacts nearly one billion adults, with a rising incidence due to the obesity epidemic (4). This epidemiological overlap underscores the critical need to explore their potential interconnection to develop effective multidisciplinary approaches in patient care (5, 6).

The pathophysiological link between both entities is primarily driven by chronic systemic inflammation and intermittent hypoxia, a central characteristic of OSA (7). Oxygen deprivation triggers the release of pro-inflammatory cytokines, such as tumor necrosis factor-alpha and interleukin-6, mediators that are fundamental in the progression of periodontal tissue destruction (1, 6). Furthermore, recurrent cycles of hypoxia-reoxygenation generate oxidative stress that exacerbates structural damage and promotes dysbiosis in the oral microbiome, creating a feedback loop that deteriorates both respiratory and periodontal health (5, 7).

Various contemporary clinical studies and meta-analyses have demonstrated a significant positive association between OSA severity and periodontal status (1). Investigations involving more than 30,000 participants report that patients with high scores on the Apnea-Hypopnea Index present greater probing depths and clinical attachment loss, with nearly twice the likelihood of suffering from periodontitis compared to healthy subjects (5, 8). This correlation, which suggests a dose-response relationship where nocturnal oxygen desaturation predicts periodontal degradation, remains statistically significant even after adjusting for fundamental demographic variables (6, 7).

In addition to systemic mechanisms, local and mechanical factors, such as mouth breathing associated with airway collapse, exacerbate gingival pathology (5). This nocturnal habit induces xerostomia, altering the protective properties of saliva and favoring the formation of pathogenic biofilms at the gingival margin (1, 6). Nevertheless, it is imperative to note that some investigations find no significant associations after rigorous control of confounding factors, suggesting that the relationship might be mediated by complex pathways where oral hygiene and biofilm play a determining role (7, 9).

Both pathologies share a complex network of common risk factors that complicate their independent study, notably obesity, smoking, aging, and hypertension (1, 10). The presence of metabolic syndrome and diabetes mellitus enhances the severity of both conditions, linking this comorbidity with a notable increase in the risk of cardiovascular diseases (5, 7). The convergence of these variables highlights the systemic nature of both diseases and demands rigorous statistical analysis to isolate the direct effect of OSA on dental support structures (6).

In the last decade, the proliferation of systematic reviews has attempted to consolidate this evidence by synthesizing data from cross-sectional studies in various geographical regions (6, 7). However, current secondary literature is heterogeneous and varies significantly in its inclusion criteria and quality assessment methods (1). While some works confirm a robust relationship, others find weaker associations when controlling for body mass index, resulting in a fragmentation of evidence that hinders clinical decision-making based on solid proof (5, 7).

Despite the growing body of literature, a critical gap persists due to the lack of standardized diagnostic protocols and the absence of robust longitudinal studies (7, 11). The available evidence does not clearly discern whether the relationship is causal, bidirectional, or simply a reflection of shared risks, which limits the implementation of integrated clinical practice guidelines (6). Consequently, there is an imperative need to conduct an umbrella review to evaluate the quality and consistency of existing systematic reviews, providing an essential third-level synthesis for researchers and clinicians (5, 6).

This umbrella review aims to comprehensively synthesize and evaluate current evidence on the association between periodontal disease and obstructive sleep apnea. By assessing available systematic reviews and meta-analyses, this study seeks to clarify the strength of the link, identify methodological limitations, and propose directions for future research. Ultimately, this manuscript intends to provide a consolidated perspective to inform clinical practice and establish a robust scientific basis for managing these two significant comorbid health conditions.

## 2 Materials and methods

### 2.1 Protocol and registration

This umbrella review was conducted following the Preferred Reporting Items for Systematic Review and Meta-Analysis Protocols (PRISMA-P) guidelines (12), and was registered in the Open Science Framework (OSF) under the DOI number DOI 10.17605/OSF.IO/RMQWC (https://archive.org/details/osf-registrations-rmqwc-v1), which is publicly accessible. Furthermore, the reporting of this review adhered to the PRIO-harms checklist (Preferred Reporting Items for Overviews of Systematic Reviews) (13). Given the nature of the study, ethical approval was not required.

### 2.2 Eligibility criteria and results of interest

Studies eligible for inclusion were systematic reviews (with or without meta-analysis) assessing primary research determining the association between PD and OSA. No restrictions were applied regarding publication date or language. Excluded publication types were narrative reviews, rapid reviews, interventional studies, observational research, preclinical and basic science studies, protocols, abstracts, case reports, commentaries, letters, opinions, and poster presentations.

### 2.3 Sources of information, search strategy and additional search for primary studies

An electronic literature search was conducted on December 27, 2025, using four major databases: PubMed, Cochrane Library, Scopus, Web of Science, Embase and SciELO. To identify additional records, gray literature sources were also consulted, including Google Scholar, ProQuest Dissertations and Theses, and OpenAIRE. Reference lists of included studies were manually screened to identify any relevant additional publications. All retrieved articles were imported into Zotero® (Center for History and New Media, Virginia, USA), and duplicates were removed. Detailed search strategies for each database are presented in Supplementary Material 1.

### 2.4 Data management and selection process

The screening and selection process was carried out using Rayyan® online software (Qatar Computing Research Institute, Qatar). Study selection was conducted in two phases. In the first phase, two independent reviewers (CAF-G and PMH-P) assessed titles and abstracts. In the second phase, the full texts of potentially relevant studies were evaluated independently by the same reviewers. Disagreements at any stage were resolved through discussion with a third reviewer (HIA-V).

### 2.5 Data collection process

Data extraction was carried out independently and in duplicate by two reviewers (TER-C and BQ-Q) using a standardized data collection form. Extracted information was cross-checked for consistency, and disagreements were resolved by a third author (HIA-V). The following variables were recorded: author names, year of publication, type of systematic review, characteristics of included primary studies, number of studies included in qualitative and quantitative analyses, reported outcomes, main conclusions, and whether the reviews reported adherence to PRISMA guidelines, PROSPERO registration or another public platform, use of the GRADE system, and performance of a meta-analysis.

### 2.6 Assessment of methodological quality, quality of evidence and meta-bias

The methodological quality and meta-bias or the risk of bias of the included systematic reviews was assessed independently and in duplicate by two reviewers (FTC-Z and RA-I), calibrated (Kappa 0.86), using the AMSTAR-2 checklist (A MeaSurement Tool to Assess Systemic Reviews) (14) and the ROBIS tool (15). The overall confidence level in the studies was rated as high, moderate, low, or critically low, and the risk of bias was rated as high, unclear or low.

### 2.7 Summary of measures

For systematic reviews (SRs) that did not include a meta-analysis, the extracted outcomes were reported by determining the association between PD and OSA. In cases where the SRs provided a meta-analysis, the results were recorded using either odss ratio (OR) or standardized mean difference (SMD).

### 2.8 Summary of results

The primary findings from the included systematic reviews were organized and reported according to key outcomes including: general association, progression and severity of PD, continent and periodontal clinical parameters (probing depth, clinical attachment loss, bleeding on probing, plaque index and gingival index).

## 3 Results

### 3.1 Review and selection of primary studies

**Figure 1:**
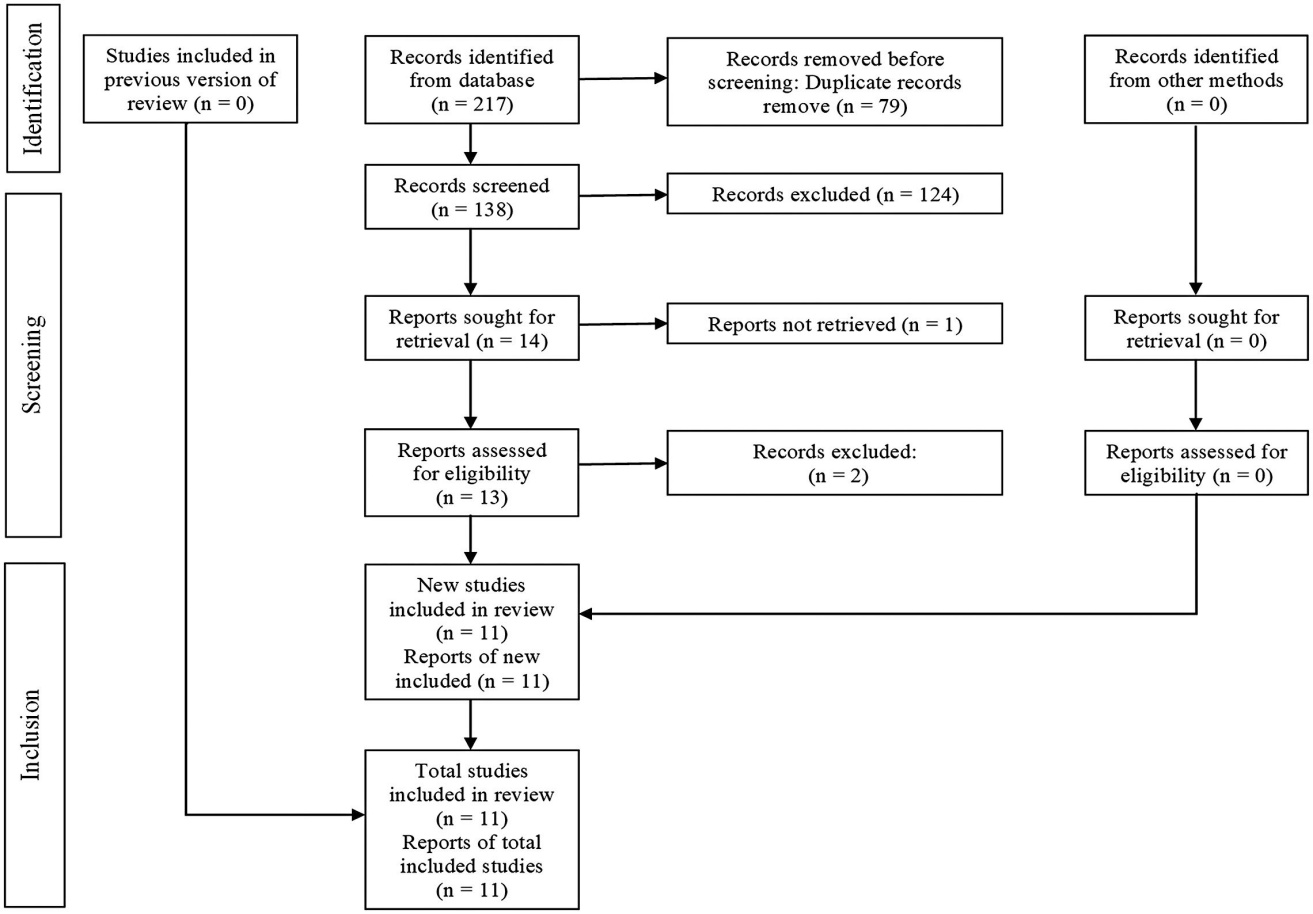
PRISMA flow diagram showing the selection process of studies included in the systematic review, from initial identification to final inclusion.

A total of 217 records were identified through the electronic database search. After eliminating duplicates, 138 unique references remained. In the initial screening phase, titles and abstracts were reviewed, resulting in 13 studies deemed suitable for full-text assessment. Of these, 2 studies were excluded, leaving 11 studies for analysis. Details regarding the exclusion criteria applied during the selection process are provided in Supplementary Material 2. The complete workflow for study identification and selection is illustrated in Figure 1.

### 3.2 Review and characteristics of included studies

The included SRs were published between 2015 and 2024. The studies originated from a diverse range of countries, including Italy (11, 16, 17), Spain (18), France (18), China (6, 8, 19), Portugal (20), Iran (2), United State (1, 21), Greece (1) and Saudi Arabia (21). Further details regarding the characteristics of the SRs are provided in Supplementary Material 3.

### 3.3 Assessment of methodological quality, quality of evidence and meta-bias

5 SRs were considered to have high confidence (1, 6, 18, 20, 21), 1 SR had low confidence (19) and 5 SRs had critically low confidence (2, 8, 11, 16, 17) (Supplementary Material 4). Furthermore, 1 SR was found to have a high risk of bias (11) and 10 SRs were found to have a low risk of bias (1, 2, 6, 8, 16–21) (Supplementary Material 3).

To mitigate the impact of low-confidence reviews on our overall findings, a sensitivity analysis was conducted, excluding studies with low or critically low confidence, and high risk of bias. The results presented here are based exclusively on high-confidence and low risk of bias reviews, ensuring the reliability of our conclusions.

### 3.4 Overlapping

A total of 113 primary studies were identified within the SRs. The degree of overlap according to the CCA index is 33.46%, and this value indicates “very high overlap”. Specifically, 4 studies were included twice, 1 appeared three times, 4 were included four times, 2 appeared five times, 2 were included six times, 2 appeared seven times, 1 were included ten times and 3 studies were included eleven times. Further details on the degree of overlap and characteristics of the primary studies are provided in Supplementary Material 5.

### 3.5 Synthesis of results

The synthesis of the results is presented in Supplementary Material 3.

### 3.6 Overall results

#### 3.6.1 General association

Eleven SRs (1, 2, 6, 8, 11, 16–21) included reported that there is an association between PD and OSA. Eight SRs (1, 2, 6, 8, 16, 18, 19, 21) meta-analyzed the results, where they found that the OR ranged from 1.56 [CI: 1.06–2.06] (6) to 2.46 [CI: 1.73–3.49] (16).

Bianchi et al. (11) reported that 11 of the 14 included studies reported a significant positive relationship between PD and OSA. Rocha Rodriguez et al. (20) reported prevalence of periodontitis ranged between 17.5% and 77% to 96.4% in patients with OSA. Lembo et al. (17) reported that the individual studies reported an OR for periodontitis in subjects with OSA that ranged from 1.37 to 1.84.

#### 3.6.2 Progression and severity of PD

Two SRs (2, 19) reported that there is an association between the progression and severity of PD and OSA. These studies meta-analyzed their results and found that the OR ranged from 1.39 [CI: 1.20–1.61] (19) to 2.51 [CI: 1.32–4.78] (2) for severe periodontitis and mild to moderate periodontitis, respectively.

#### 3.6.3 Continent

One SR (19) reported an association between PD and OSA in Asian and non-Asian populations. This study meta-analyzed its results and found that the OR was 2.10 [CI: 1.57–2.80] and 1.51 [CI: 1.21–1.88] for Asian and non-Asian individuals, respectively.

#### 3.6.4 Periodontal clinical parameters

One SR (8) reported an association between PD and OSA. This study meta-analyzed its results and found that the SMD was 0.68 [CI: 0.06–1.30], 0.69 [CI: 0.17–1.22] and 0.36 [CI: 0.08–0.64] for probing depth (Pd), clinical attachment loos (CAL) and bleeding on probing (BOP), respectively.

One SR (8) reported no association between PD and OSA. This study meta-analyzed its results and found that the SMD was 0.11 [CI: −0.18–0.39] and 0.18 [CI: −0.18–0.53] for plaque and gingival index, respectively.

### 3.7 Sensitivity analysis of the results

#### 3.7.1 General association

Five SRs (1, 6, 18, 20, 21) included reported that there is an association between PD and OSA. Four SRs (1, 6, 18, 21) meta-analyzed the results, where they found that the OR ranged from 1.56 [CI: 1.06–2.06] (6) to 1.66 [CI: 1.28–2.17] (1). Rocha Rodriguez et al. (20) reported prevalence of periodontitis ranged between 17.5% and 77% to 96.4% in patients with OSA.

## 4 Discussion

The present umbrella review synthesizes evidence indicating a consistent positive association between PD and OSA. This interconnection suggests that chronic systemic inflammation, a hallmark of sleep-disordered breathing, may be associated with periodontal deterioration through shared inflammatory pathways. From a clinical perspective, the consistency of these results reinforces the idea that OSA may represent a clinical condition associated with increased periodontal burden, supporting closer surveillance and diagnostic integration between sleep medicine and dentistry.

The reported evidence aligns with recent meta-analyses documenting a positive association (2, 8, 11, 16, 19). Some findings indicate that nearly 96.4% of patients with OSA have a substantially higher probability of suffering from periodontitis, with a prevalence that can reach up to 77% (20). It is particularly relevant that this association intensifies according to the severity of PD; this could be explained by common factors shared between both pathologies, such as chronic systemic inflammation, mouth breathing, or the presence of comorbidities (16). Furthermore, a notable geographic disparity has been observed, where Asian populations show a higher risk compared to non-Asian populations, which could be attributed to genetic variations, specific craniofacial phenotypes, and differences in regional diagnostic thresholds.

Regarding clinical parameters, the increase in Pd, CAL and BOP in patients with OSA supports the observation that OSA is associated with more severe periodontal parameters. The effect size found for probing depth and attachment loss is categorized as a moderate-to-strong effect, indicating that the difference in periodontal destruction between patients with and without OSA may be clinically relevant. However, discrepancies exist in the literature; some studies found no significant correlations with the apnea-hypopnea index (AHI) (9) or concluded that the evidence is still scarce and of low quality (17). These inconsistencies usually derive from methodological limitations, such as small sample sizes or the lack of control for confounders like smoking and obesity (9, 22). Despite this, research such as that by Chen et al. (23) suggests that while plaque is the primary factor, the altered host response in OSA patients favors disproportionate tissue destruction.

Although the primary evidence is observational and does not establish causality, several biological mechanisms have been proposed to explain the observed association. The pathophysiological mechanisms underlying this relationship are multidimensional. OSA induces episodes of hypoxemia and reoxygenation that activate oxidative stress pathways and elevate systemic proinflammatory markers such as IL-1β, IL-6, TNF-α, and C-reactive protein (CRP) (16, 22). These substances, detected in both serum and crevicular fluid, promote the degradation of periodontal tissue (7). Simultaneously, chronic mouth breathing induces xerostomia, which alters the oral microbiome and favors the accumulation of dysbiotic bacterial plaque (7, 16). This cycle of reciprocal inflammation is exacerbated by shared risk factors, such as diabetes and obesity, which act synergistically in both pathologies (7, 20).

From a methodological standpoint, this review presents strengths and limitations inherent to its design. A high overlap of primary studies was identified (CCA: 33.46%), a common phenomenon in umbrella reviews that could bias global concordance if not critically addressed. To mitigate this, a sensitivity analysis focusing on high-confidence reviews was conducted. Most of these reported adjusted odds ratios (ORs) with consistent estimates between 1.56 and 1.66. Despite the high overlap, the stability of results across different inclusion criteria (e.g., polysomnography vs. questionnaires) and adjustment for confounders such as age, obesity, BMI, and smoking indicates that the observed association remains consistent across methodological variations, although causality cannot be inferred. Nevertheless, considerable heterogeneity exists in the diagnostic criteria used for both periodontal disease and obstructive sleep apnea across the included systematic reviews. Periodontal disease definitions varied according to clinical attachment level (CAL) thresholds, probing depth criteria, or CDC-AAP classifications, while OSA diagnosis ranged from polysomnography-based AHI measurements to questionnaire-based screening tools with different cut-off values. This variability may influence both prevalence estimates and the magnitude of the reported associations. Although most reviews reported consistent positive associations regardless of diagnostic approach, the absence of standardized definitions limits direct comparability across studies. Future research would benefit from stratified analyzes based on diagnostic methodology and the adoption of harmonized diagnostic criteria to enhance interpretability and reduce methodological heterogeneity.

It is important to emphasize that most primary studies included in the analyzed systematic reviews were cross-sectional in design. Therefore, temporality and causal direction cannot be established. The findings should be interpreted as evidence of association rather than proof of a causal or risk-factor relationship. Longitudinal and interventional studies are needed to clarify the directionality and potential bidirectional mechanisms between PD and OSA.

Finally, the clinical implications of these findings demand an interdisciplinary approach and integrated public health policies. Dentists may consider screening for signs suggestive of OSA in patients with periodontitis, while sleeping medicine or pulmonology specialists may consider periodontal evaluation in patients diagnosed with OSA. Although interventional evidence is still limited, exploring whether treatment of one condition influences the other represents an important direction for future research. In clinical practice, promoting oral hygiene and addressing modifiable risk factors (e.g., obesity, smoking) in patients with OSA may contribute to improved overall oral health. At a population level, these results highlight the importance of integrated oral and respiratory prevention policies. Ultimately, the identified association emphasizes the need to raise awareness among both patients and clinicians regarding the interaction between oral health and sleep-disordered breathing and motivates future research to clarify the causal nature of this relationship.

## 5 Conclusions

This umbrella review, based on systematic reviews with low risk of bias and high overall confidence, indicates a consistent association between PD and OSA. However, due to the predominantly observational nature of the primary evidence, causality cannot be inferred, and further longitudinal and interventional research is warranted.

## Data Availability

The original contributions presented in the study are included in the article/Supplementary Material, further inquiries can be directed to the corresponding author.

## Supplementary material

SUPPLEMENTARY MATERIAL 1

Database search strategy — A detailed description of the search terms and strategies used for each database.

SUPPLEMENTARY MATERIAL 2

Reason for exclusion of studies — A list of studies that were excluded from the review with reasons for their exclusion.

SUPPLEMENTARY MATERIAL 3

Characteristics of included studies — A table summarizing the characteristics of the systematic reviews included in the analysis.

SUPPLEMENTARY MATERIAL 4

Assessment of the methodological quality and the quality of the evidence of the included studies — A summary of the quality assessments for the included systematic reviews using the AMSTAR-2 tool.

SUPPLEMENTARY MATERIAL 5

Overlap of primary studies in systematic reviews — An overview of the overlap of primary studies across the included systematic reviews.
