# Supplementary material for: Periodontal disease and obstructive sleep apnea: an umbrella review
Source: Front Oral Health. 2026 Mar 26;7:1780859. doi: 10.3389/froh.2026.1780859 (PMC13062253; doi:10.3389/froh.2026.1780859)
Supplement: Supplementary file 1 [file Table1.docx]

Supplementary Material 1. Database search strategy

| **Database** | **Search strategy** | **Number of studies** |
| --- | --- | --- |
| Pubmed | (("Periodontal Diseases"[Mesh] OR "Periodontitis"[Mesh] OR "Gingivitis"[Mesh] OR "Alveolar Bone Loss"[Mesh] OR "periodontal disease*"[tiab] OR "periodontitis"[tiab] OR "gingivitis"[tiab] OR "gum disease*"[tiab] OR "periodontal pocket*"[tiab] OR "alveolar bone loss"[tiab] OR "tooth loss"[tiab]) AND ("Sleep Apnea, Obstructive"[Mesh] OR "obstructive sleep apnea*"[tiab] OR "OSA"[tiab] OR "OSAS"[tiab] OR "obstructive sleep hypopnea*"[tiab] OR "sleep disordered breathing"[tiab] OR "obstructive sleep apnea syndrome"[tiab]) AND ("Systematic Reviews as topic"[Mesh] OR "Meta-Analysis as topic"[Mesh] OR "systematic review*"[tiab] OR "meta-analysis"[tiab] OR "meta-analyses"[tiab])) | 21 |
| Cochrane library | ([mh "Periodontal Diseases"] OR [mh "Periodontitis"] OR [mh "Gingivitis"] OR "periodontal disease*":ti,ab,kw OR "periodontitis":ti,ab,kw OR "gingivitis":ti,ab,kw OR "gum disease*":ti,ab,kw ) AND ( [mh "Sleep Apnea, Obstructive"] OR "obstructive sleep apnea*":ti,ab,kw OR "OSA":ti,ab,kw OR "OSAS":ti,ab,kw OR "sleep disordered breathing":ti,ab,kw ) | 0 |
| Scopus | TITLE-ABS-KEY(("periodontal disease*" OR "periodontitis" OR "gingivitis" OR "gum disease*" OR "periodontal pocket*" OR "alveolar bone loss") AND ("obstructive sleep apnea*" OR "OSA" OR "OSAS" OR "obstructive sleep hypopnea*" OR "sleep disordered breathing") AND ("systematic review*" OR "meta-analysis" OR "meta-analyses")) | 36 |
| Web of Science | TS=(("periodontal disease*" OR "periodontitis" OR "gingivitis" OR "gum disease*" OR "periodontal pocket*" OR "alveolar bone loss") AND ("obstructive sleep apnea*" OR "OSA" OR "OSAS" OR "obstructive sleep hypopnea*" OR "sleep disordered breathing") AND ("systematic review*" OR "meta-analysis" OR "meta-analyses")) | 19 |
| Embase | ('periodontal disease'/exp OR 'periodontitis'/exp OR 'gingivitis'/exp OR 'periodontal disease*':ab,ti OR 'periodontitis':ab,ti OR 'gingivitis':ab,ti OR 'gum disease*':ab,ti OR 'periodontal pocket*':ab,ti OR 'alveolar bone loss':ab,ti) AND ('obstructive sleep apnea'/exp OR 'obstructive sleep apnea*':ab,ti OR 'osa':ab,ti OR 'osas':ab,ti OR 'obstructive sleep hypopnea*':ab,ti OR 'sleep disordered breathing':ab,ti) AND ('systematic review'/exp OR 'meta analysis'/exp OR 'systematic review*':ab,ti OR 'meta-analysis':ab,ti OR 'meta-analyses':ab,ti) | 25 |
| Scielo | (ti:(periodontal* OR periodontitis OR gingivitis OR "enfermedad periodontal" OR "doença periodontal") OR ab:(periodontal* OR periodontitis OR gingivitis OR "enfermedad periodontal" OR "doença periodontal")) AND (ti:("sleep apnea" OR "apnea del sueño" OR "apneia do sono" OR "obstructive sleep apnea" OR "apnea obstructiva" OR OSA) OR ab:("sleep apnea" OR "apnea del sueño" OR "apneia do sono" OR "obstructive sleep apnea" OR "apnea obstructiva" OR OSA)) AND (ti:("systematic review" OR "revisión sistemática" OR "revisão sistemática" OR "meta-analysis" OR metaanálisis OR metanálise)) | 0 |
| Google Scholar | ("periodontal disease" OR periodontitis) AND ("obstructive sleep apnea" OR OSA) AND ("systematic review" OR "meta-analysis") -"overview" -"pilot study" | 100 |
| Proquest Dissertations and Theses | (TI,AB(periodontal* OR periodontitis OR gingivitis OR "gum disease*" OR "alveolar bone loss" OR "periodontal pocket*") AND TI,AB("obstructive sleep apnea*" OR "OSA" OR "OSAS" OR "obstructive sleep hypopnea*" OR "sleep disordered breathing") AND TI,AB("systematic review*" OR "meta-analysis" OR "meta-analyses")) | 16 |
| OpenAIRE | (periodontal* OR periodontitis OR "periodontal disease") AND ("obstructive sleep apnea" OR "OSA") AND ("systematic review" OR "meta-analysis") | 0 |
