# Supplementary material for: Periodontal disease and obstructive sleep apnea: an umbrella review
Source: Front Oral Health. 2026 Mar 26;7:1780859. doi: 10.3389/froh.2026.1780859 (PMC13062253; doi:10.3389/froh.2026.1780859)
Supplement: Supplementary file 2 [file Table2.docx]

Supplementary Material 2. Reason for exclusion of studies

| **Author** | **Reason for exclusion** |
| --- | --- |
| Pachiou et al. (1) | No periodontal disease |
| de Araujo Dantas et al. (2) |  |

**References:**

1. Pachiou A, Roulias P, Steiropoulos P, Halazonetis DJ, Kourtis S. Association of edentulism and obstructive sleep apnea: A systematic review. *J Prosthodont* (2025) 34:90–99. doi: 10.1111/jopr.13981

2. de Araujo Dantas AB, Goncalves FM, Martins AA, Alves GÂ, Stechman-Neto J, Correa C de C, Santos RS, Nascimento WV, de Araujo CM, Taveira KVM. Worldwide prevalence and associated risk factors of obstructive sleep apnea: a meta-analysis and meta-regression. *Sleep and Breathing* (2023) 27:2083–2109.
