# Supplementary material for: Periodontal disease and obstructive sleep apnea: an umbrella review
Source: Front Oral Health. 2026 Mar 26;7:1780859. doi: 10.3389/froh.2026.1780859 (PMC13062253; doi:10.3389/froh.2026.1780859)
Supplement: Supplementary file 3 [file Table3.docx]

Supplementary Material 3. Characteristics of included studies

| **Authors** | **Year** | **Study design** | **Country** | **Included study design** | **Number of studies (qualitative / quantitative)** | **Register** | **PRISMA** | **GRADE** | **ROBIS** | **Outcomes** | | **Conclusions** |  |
| --- | --- | --- | --- | --- | --- | --- | --- | --- | --- | --- | --- | --- | --- |
| Portelli et al. (1) | 2024 | SR and MA | Italy | CC and CS | 10/10 | Yes | Yes | No | Low | General | OR = 2.46 (1.73 – 3.49) | There is a statistically significant association between OSAS and periodontitis. | |
| Bianchi et al. (2) | 2024 | SR | Italy | CC and CS | 14/0 | No | Yes | No | High | General | 11 of the 14 studies reported a significant positive relationship between PD and OSA. | There is evidence of a plausible association between periodontitis and OSA. | |
| Molina et al. (3) | 2023 | SR and MA | Spain and France | CC and CS | 12/6 | Yes | Yes | No | Low | General | OR = 1.65 (1.21 – 2.25)* | Periodontitis is significantly associated with OSA. | |
| Liu et al. (4) | 2023 | SR and MA | China | CC and CS | 10/10 | No | Yes | No | Low | General | OR = 1.83 (1.52 – 2.20) | A PSG-confirmed diagnosis of SDB is significantly associated with a higher likelihood of periodontitis in adults. | |
|  |  |  |  |  |  |  |  |  |  | General | OR = 1.68 (1.47 – 1.92)* |  |  |
|  |  |  |  |  |  |  |  |  |  | Severe periodontitis | OR = 1.39 (1.20 – 1.61)* |  |  |
|  |  |  |  |  |  |  |  |  |  | Asian | OR =2.10 (1.57 – 2.80) |  |  |
|  |  |  |  |  |  |  |  |  |  | Non-Asian | OR = 1.51 (1.21 – 1.88) |  |  |
| Zhu et al. (5) | 2023 | SR and MA | China | CC and CS | 13/13 | No | Yes | No | Low | General | OR = 2.35 (2.22 – 2.48) | OSA is significantly associated with a higher prevalence of periodontitis and a deteriorated periodontal condition, manifested in higher levels of PD and CAL. | |
|  |  |  |  |  |  |  |  |  |  | Pd | SMD = 0.68 (0.06 – 1.30) |  |  |
|  |  |  |  |  |  |  |  |  |  | CAL | SMD = 0.69 (0.17 – 1.22) |  |  |
|  |  |  |  |  |  |  |  |  |  | BOP | SMD = 0.36 (0.08 – 0.64) |  |  |
|  |  |  |  |  |  |  |  |  |  | PI | SMD = 0.11 (-0.18 – 0.39) |  |  |
|  |  |  |  |  |  |  |  |  |  | GI | SMD = 0.18 (-0.18 – 0.53) |  |  |
| Rocha Rodrigues et al. (6) | 2023 | SR | Portugal | CC and CS | 6/0 | Yes | No | No | Low | General | The reported prevalence of periodontitis ranged between 17.5% and  77% to 96.4% in patients with OSA. | Most studies suggest a positive association between periodontitis and OSA. | |
| Zhang et al. (7) | 2022 | SR and MA | China | CC and CS | 9/6 | Yes | Yes | No | Low | General | OR = 1.56 (1.06 – 2.06) | OSA is associated with a statistically significant increase in the risk of periodontitis. | |
| Khodadadi et al. (8) | 2022 | SR and MA | Iran | CC and CS | 10/10 | No | Yes | No | Low | General | OR = 2.17 (1.66 – 2.83) | There is a direct and significant association between periodontitis and OSA. | |
|  |  |  |  |  |  |  |  |  |  | General | OR = 1.75 (1.65 – 1.85)* |  |  |
|  |  |  |  |  |  |  |  |  |  | Mild - Moderate periodontitis | OR = 2.51 (1.32 – 4.78) |  |  |
| Lembo et al. (9) | 2021 | SR | Italy | CC and CS | 10/0 | No | Yes | No | Low | General | Individual studies reported an OR for periodontitis in subjects with OSA that ranged from 1.37 to 1.84. | There is a possible association between OSA and periodontitis, but the evidence is weak. | |
| Al-Jewair et al. (10) | 2020 | SR and MA | United States and Greece | CC and CS | 13/9 | Yes | Yes | Yes | Low | General | OR = 1.66 (1.28 - 2.17)* | There is a significant positive association between periodontitis and OSA. | |
| Al-Jewair et al. (11) | 2015 | SR and MA | Saudi Arabia and United States | CC and CS | 6/4 | Yes | Yes | No | Low | General | OR = 1.65 (1.11 - 2.46)* | There is evidence of a plausible association between PD and OSA. | |

SR = Systematic review; MA = Meta-analysis; CC = Case and control; CS = Cross-sectional study; PD = Periodontal disease; OSA = Obstructive sleep apnea; PSG = Polysomnography; SBD = Sleep-disordered breathing; Pd = Probing depth; CAL = Clinical attachment loss; BOP = Bleeding on probing; PI = Plaque index; GI = Gingival index; OR = Odds ratio; SMD = Standardized mean difference; *= Adjusted model

**References**

1. Portelli M, Russo I, Bellocchio AM, Militi A, Nucera R. Correlations between Obstructive Sleep Apnea Syndrome and Periodontitis: A Systematic Review and Meta-Analysis. *Dent J (Basel)* (2024) 12: doi: 10.3390/dj12080236

2. Bianchi E, Segù M, Toffoli A, Razzini G, Macaluso GM, Manfredi E. Relationship between periodontal disease and obstructive sleep apnea in adults: A systematic review. *Dent Res J (Isfahan)* (2024) 21:15.

3. Molina A, Huck O, Herrera D, Montero E. The association between respiratory diseases and periodontitis: A systematic review and meta-analysis. *J Clin Periodontol* (2023) 50:842–887. doi: 10.1111/jcpe.13767

4. Liu X, Zhu Z, Zhang P. Association between sleep-disordered breathing and periodontitis: a meta-analysis. *Med Oral Patol Oral Cir Bucal* (2023) 28:e156–e166. doi: 10.4317/medoral.25627

5. Zhu J, Yuan X, Zhang Y, Wei F, Hou Y, Zhang Y. A meta-analysis on the association between obstructive sleep apnea and periodontitis. *Sleep Breath* (2023) 27:641–649. doi: 10.1007/s11325-022-02668-1

6. Rocha Rodrigues V, Falardo Ramos S. Is there an association with periodontitis and obstructive sleep apnea? A systematic review. *J Dent Sleep Med* (2023) 10:1–15. doi: 10.15331/jdsm.7278

7. Zhang Z, Ge S, Zhai G, Yu S, Cui Z, Si S, Chou X. Incidence and risk of periodontitis in obstructive sleep apnea: A meta-analysis. *PLoS One* (2022) 17:e0271738. doi: 10.1371/journal.pone.0271738

8. Khodadadi N, Khodadadi M, Zamani M. Is periodontitis associated with obstructive sleep apnea? A systematic review and meta-analysis. *J Clin Exp Dent* (2022) 14:e359–e365. doi: 10.4317/jced.59478

9. Lembo D, Caroccia F, Lopes C, Moscagiuri F, Sinjari B, D’Attilio M. Obstructive Sleep Apnea and Periodontal Disease: A Systematic Review. *Medicina (Kaunas)* (2021) 57:640. doi: 10.3390/medicina57060640

10. Al-Jewair T, Apessos I, Stellrecht E, Koch R, Almaghrabi B. An Update on the Association Between Periodontitis and Obstructive Sleep Apnea. *Cur Oral Heal Rep* (2020) 7:189–201. doi: 10.1007/s40496-020-00271-5

11. Al-Jewair TS, Al-Jasser R, Almas K. Periodontitis and obstructive sleep apnea’s bidirectional relationship: a systematic review and meta-analysis. *Sleep Breath* (2015) 19:1111–1120. doi: 10.1007/s11325-015-1160-8
