# Supplementary material for: Periodontal disease and obstructive sleep apnea: an umbrella review
Source: Front Oral Health. 2026 Mar 26;7:1780859. doi: 10.3389/froh.2026.1780859 (PMC13062253; doi:10.3389/froh.2026.1780859)
Supplement: Supplementary file 4 [file Table4.docx]

Supplementary Material 4. Assessment of the methodological quality and the quality of the evidence of the included studies

| Authors | Year | AMSTAR – 2 | | | | | | | | | | | | | | | | Overall confidence |
| --- | --- | --- | --- | --- | --- | --- | --- | --- | --- | --- | --- | --- | --- | --- | --- | --- | --- | --- |
|  |  | 1 | 2* | 3 | 4* | 5 | 6 | 7* | 8 | 9* | 10 | 11* | 12 | 13* | 14 | 15* | 16 |  |
| Portelli et al. (1) | 2024 | Yes | Yes | Yes | Yes | No | Yes | No | Yes | Yes | No | Yes | No | No | Yes | Yes | Yes | Critically low |
| Bianchi et al. (2) | 2024 | Yes | No | Yes | No | Yes | Yes | No | Yes | Yes | No | No meta-analysis | | Yes | Yes | No meta-analysis | Yes | Critically low |
| Molina et al. (3) | 2023 | Yes | Yes | Yes | Yes | Yes | No | Yes | Yes | Yes | Yes | Yes | Yes | Yes | Yes | Yes | Yes | High |
| Liu et al. (4) | 2023 | Yes | No | Yes | Yes | Yes | Yes | Yes partial | Yes | Yes | No | Yes | Yes | Yes | Yes | Yes | Yes | Low |
| Zhu et al. (5) | 2023 | Yes | No | Yes | Yes | Yes | Yes | Yes partial | Yes | Yes | No | Yes | Yes | Yes | Yes | No | Yes | Critically low |
| Rocha Rodrigues et al. (6) | 2023 | Yes | Yes | Yes | Yes | Yes | Yes | Yes | Yes | Yes | No | No meta-analysis | | Yes | Yes | No meta-analysis | Yes | High |
| Zhang et al. (7) | 2022 | Yes | Yes | Yes | Yes | Yes | Yes | Yes partial | Yes | Yes | No | Yes | Yes | Yes | Yes | Yes | Yes | High |
| Khodadadi et al. (8) | 2022 | Yes | No | Yes | Yes | Yes | Yes | Yes partial | Yes | Yes | No | Yes | Yes | No | Yes | Yes | Yes | Critically low |
| Lembo et al. (9) | 2021 | Yes | No | Yes | Yes | Yes | Yes | No | Yes | Yes | No | No meta-analysis | | Yes | Yes | No meta-analysis | Yes | Critically low |
| Al-Jewair et al. (10) | 2020 | Yes | Yes | Yes | Yes | Yes | Yes | Yes | Yes | Yes | No | Yes | Yes | Yes | Yes | Yes | Yes | High |
| Al-Jewair et al. (11) | 2015 | Yes | Yes | Yes | Yes | Yes | Yes | Yes | Yes | Yes | No | Yes | Yes | Yes | Yes | Yes | Yes | High |

AMSTAR = A MeaSurement Tool to Assess Systemic Reviews

1 = Did the research questions and inclusion criteria for the review include the components of PICO?

2 = Did the report of the review contain an explicit statement that the review methods were established prior to the conduct of the review and did the report justify any significant deviations from the protocol?

3 = Did the review authors explain their selection of the study designs for inclusion in the review?

4 = Did the review authors use a comprehensive literature search strategy?

5 = Did the review authors perform study selection in duplicate?

6 = Did the review authors perform data extraction in duplicate?

7 = Did the review authors provide a list of excluded studies and justify the exclusions?

8 = Did the review authors describe the included studies in adequate detail?

9 = Did the review authors use a satisfactory technique for assessing the risk of bias (RoB) in individual studies that were included in the review?

10 = Did the review authors report on the sources of funding for the studies included in the review?

11 = If meta-analysis was performed, did the review authors use appropriate methods for statistical combination of results?

12 = If meta-analysis was performed, did the review authors assess the potential impact of RoB in individual studies on the results of the meta-analysis or other evidence synthesis?

13 = Did the review authors account for RoB in primary studies when interpreting/discussing the results of the review?

14 = Did the review authors provide a satisfactory explanation for, and discussion of, any heterogeneity observed in the results of the review?

15 = If they performed quantitative synthesis did the review authors carry out an adequate investigation of publication bias (small study bias) and discuss its likely impact on the results of the review?

16 = Did the review authors report any potential sources of conflict of interest, including any funding they received for conducting the review?

* = Critical domain

**References**

1. Portelli M, Russo I, Bellocchio AM, Militi A, Nucera R. Correlations between Obstructive Sleep Apnea Syndrome and Periodontitis: A Systematic Review and Meta-Analysis. *Dent J (Basel)* (2024) 12: doi: 10.3390/dj12080236

2. Bianchi E, Segù M, Toffoli A, Razzini G, Macaluso GM, Manfredi E. Relationship between periodontal disease and obstructive sleep apnea in adults: A systematic review. *Dent Res J (Isfahan)* (2024) 21:15.

3. Molina A, Huck O, Herrera D, Montero E. The association between respiratory diseases and periodontitis: A systematic review and meta-analysis. *J Clin Periodontol* (2023) 50:842–887. doi: 10.1111/jcpe.13767

4. Liu X, Zhu Z, Zhang P. Association between sleep-disordered breathing and periodontitis: a meta-analysis. *Med Oral Patol Oral Cir Bucal* (2023) 28:e156–e166. doi: 10.4317/medoral.25627

5. Zhu J, Yuan X, Zhang Y, Wei F, Hou Y, Zhang Y. A meta-analysis on the association between obstructive sleep apnea and periodontitis. *Sleep Breath* (2023) 27:641–649. doi: 10.1007/s11325-022-02668-1

6. Rocha Rodrigues V, Falardo Ramos S. Is there an association with periodontitis and obstructive sleep apnea? A systematic review. *J Dent Sleep Med* (2023) 10:1–15. doi: 10.15331/jdsm.7278

7. Zhang Z, Ge S, Zhai G, Yu S, Cui Z, Si S, Chou X. Incidence and risk of periodontitis in obstructive sleep apnea: A meta-analysis. *PLoS One* (2022) 17:e0271738. doi: 10.1371/journal.pone.0271738

8. Khodadadi N, Khodadadi M, Zamani M. Is periodontitis associated with obstructive sleep apnea? A systematic review and meta-analysis. *J Clin Exp Dent* (2022) 14:e359–e365. doi: 10.4317/jced.59478

9. Lembo D, Caroccia F, Lopes C, Moscagiuri F, Sinjari B, D’Attilio M. Obstructive Sleep Apnea and Periodontal Disease: A Systematic Review. *Medicina (Kaunas)* (2021) 57:640. doi: 10.3390/medicina57060640

10. Al-Jewair T, Apessos I, Stellrecht E, Koch R, Almaghrabi B. An Update on the Association Between Periodontitis and Obstructive Sleep Apnea. *Cur Oral Heal Rep* (2020) 7:189–201. doi: 10.1007/s40496-020-00271-5

11. Al-Jewair TS, Al-Jasser R, Almas K. Periodontitis and obstructive sleep apnea’s bidirectional relationship: a systematic review and meta-analysis. *Sleep Breath* (2015) 19:1111–1120. doi: 10.1007/s11325-015-1160-8
