# Supplementary material for: Periodontal disease and obstructive sleep apnea: an umbrella review
Source: Front Oral Health. 2026 Mar 26;7:1780859. doi: 10.3389/froh.2026.1780859 (PMC13062253; doi:10.3389/froh.2026.1780859)
Supplement: Supplementary file 5 [file Table5.docx]

Supplementary Material 5. Overlapping of primary studies in systematic reviews

| **Primary studies** | **Systematic reviews that included the primary studies** | **Times that primary studies were included** |
| --- | --- | --- |
| Keller et al. (1) | Portelli et al. (2), Bianchi et al. (3), Molina et al. (4), Liu et al. (5), Zhu et al. (6), Rocha Rodrigues et al. (7), Zhang et al. (8), Khodadadi et al. (9), Lembo et al. (10), Al-Jewair et al. (11), Al-Jewair et al. (12) | 11 |
| Loke et al. (13) | Portelli et al. (2), Bianchi et al. (3), Molina et al. (4), Liu et al. (5), Zhu et al. (6), Rocha Rodrigues et al. (7), Zhang et al. (8), Khodadadi et al. (9), Lembo et al. (10), Al-Jewair et al. (11), Al-Jewair et al. (12) | 11 |
| Seo et al. (14) | Portelli et al. (2), Bianchi et al. (3), Molina et al. (4), Liu et al. (5), Zhu et al. (6), Rocha Rodrigues et al. (7), Zhang et al. (8), Khodadadi et al. (9), Lembo et al. (10), Al-Jewair et al. (11), Al-Jewair et al. (12) | 11 |
| Gamsiz-Isik et al. (15) | Portelli et al. (2), Bianchi et al. (3), Molina et al. (4), Liu et al. (5), Zhu et al. (6), Rocha Rodrigues et al. (7), Zhang et al. (8), Khodadadi et al. (9), Lembo et al. (10), Al-Jewair et al. (11) | 10 |
| Nizam et al. (16) | Bianchi et al. (3), Molina et al. (4), Liu et al. (5), Zhu et al. (6), Rocha Rodrigues et al. (7), Lembo et al. (10), Al-Jewair et al. (11) | 7 |
| Latorre et al. (17) | Bianchi et al. (3), Liu et al. (5), Zhu et al. (6), Zhang et al. (8), Khodadadi et al. (9), Lembo et al. (10), Al-Jewair et al. (11) | 7 |
| Ahmad et al. (18) | Bianchi et al. (3), Molina et al. (4), Zhu et al. (6), Zhang et al. (8), Al-Jewair et al. (11), Al-Jewair et al. (12) | 6 |
| Sanders et al. (19) | Bianchi et al. (3), Molina et al. (4), Liu et al. (5), Zhang et al. (8), Lembo et al. (10), Al-Jewair et al. (11) | 6 |
| Sales-Peres et al. (20) | Bianchi et al. (3), Zhu et al. (6), Zhang et al. (8), Khodadadi et al. (9), Al-Jewair et al. (11) | 5 |
| Gunaratnam et al. (21) | Bianchi et al. (3), Rocha Rodrigues et al. (7), Lembo et al. (10), Al-Jewair et al. (11), Al-Jewair et al. (12) | 5 |
| Chen et al. (22) | Portelli et al. (2), Bianchi et al. (3), Liu et al. (5), Khodadadi et al. (9) | 4 |
| Nizam et al. (23) | Bianchi et al. (3), Molina et al. (4), Lembo et al. (10), Al-Jewair et al. (11) | 4 |
| Pico-Orozco et al. (24) | Portelli et al. (2), Molina et al. (4), Liu et al. (5), Khodadadi et al. (9) | 4 |
| Nizam et al. (25) | Bianchi et al. (3), Lembo et al. (10), Al-Jewair et al. (11), Al-Jewair et al. (12) | 4 |
| Al Habashneh et al. (26) | Bianchi et al. (3), Molina et al. (4), Zhu et al. (6) | 3 |
| Tamasas et al. (27) | Liu et al. (5), Zhu et al. (6) | 2 |
| Mukherjee et al. (28) | Molina et al. (4), Zhang et al. (8) | 2 |
| Chen et al. (29) | Portelli et al. (2), Khodadadi et al. (9) | 2 |
| Kale et al. (30) | Zhu et al. (6), Khodadadi et al. (9) | 2 |

**References**

1. Keller JJ, Wu C-S, Chen Y-H, Lin H-C. Association between obstructive sleep apnoea and chronic periodontitis: a population-based study. *J Clin Periodontol* (2013) 40:111–117. doi: 10.1111/jcpe.12036

2. Portelli M, Russo I, Bellocchio AM, Militi A, Nucera R. Correlations between Obstructive Sleep Apnea Syndrome and Periodontitis: A Systematic Review and Meta-Analysis. *Dent J (Basel)* (2024) 12: doi: 10.3390/dj12080236

3. Bianchi E, Segù M, Toffoli A, Razzini G, Macaluso GM, Manfredi E. Relationship between periodontal disease and obstructive sleep apnea in adults: A systematic review. *Dent Res J (Isfahan)* (2024) 21:15.

4. Molina A, Huck O, Herrera D, Montero E. The association between respiratory diseases and periodontitis: A systematic review and meta-analysis. *J Clin Periodontol* (2023) 50:842–887. doi: 10.1111/jcpe.13767

5. Liu X, Zhu Z, Zhang P. Association between sleep-disordered breathing and periodontitis: a meta-analysis. *Med Oral Patol Oral Cir Bucal* (2023) 28:e156–e166. doi: 10.4317/medoral.25627

6. Zhu J, Yuan X, Zhang Y, Wei F, Hou Y, Zhang Y. A meta-analysis on the association between obstructive sleep apnea and periodontitis. *Sleep Breath* (2023) 27:641–649. doi: 10.1007/s11325-022-02668-1

7. Rocha Rodrigues V, Falardo Ramos S. Is there an association with periodontitis and obstructive sleep apnea? A systematic review. *J Dent Sleep Med* (2023) 10:1–15. doi: 10.15331/jdsm.7278

8. Zhang Z, Ge S, Zhai G, Yu S, Cui Z, Si S, Chou X. Incidence and risk of periodontitis in obstructive sleep apnea: A meta-analysis. *PLoS One* (2022) 17:e0271738. doi: 10.1371/journal.pone.0271738

9. Khodadadi N, Khodadadi M, Zamani M. Is periodontitis associated with obstructive sleep apnea? A systematic review and meta-analysis. *J Clin Exp Dent* (2022) 14:e359–e365. doi: 10.4317/jced.59478

10. Lembo D, Caroccia F, Lopes C, Moscagiuri F, Sinjari B, D’Attilio M. Obstructive Sleep Apnea and Periodontal Disease: A Systematic Review. *Medicina (Kaunas)* (2021) 57:640. doi: 10.3390/medicina57060640

11. Al-Jewair T, Apessos I, Stellrecht E, Koch R, Almaghrabi B. An Update on the Association Between Periodontitis and Obstructive Sleep Apnea. *Cur Oral Heal Rep* (2020) 7:189–201. doi: 10.1007/s40496-020-00271-5

12. Al-Jewair TS, Al-Jasser R, Almas K. Periodontitis and obstructive sleep apnea’s bidirectional relationship: a systematic review and meta-analysis. *Sleep Breath* (2015) 19:1111–1120. doi: 10.1007/s11325-015-1160-8

13. Loke W, Girvan T, Ingmundson P, Verrett R, Schoolfield J, Mealey BL. Investigating the association between obstructive sleep apnea and periodontitis. *J Periodontol* (2015) 86:232–243. doi: 10.1902/jop.2014.140229

14. Seo WH, Cho ER, Thomas RJ, An S-Y, Ryu JJ, Kim H, Shin C. The association between periodontitis and obstructive sleep apnea: a preliminary study. *J Periodontal Res* (2013) 48:500–506. doi: 10.1111/jre.12032

15. Gamsiz‐Isik H, Kiyan E, Bingol Z, Baser U, Ademoglu E, Yalcin F. Does obstructive sleep apnea increase the risk for periodontal disease? A case‐control study. *J Periodontol* (2017) 88:443–449. doi: 10.1902/jop.2016.160365

16. Nizam N, Basoglu OK, Tasbakan MS, Lappin DF, Buduneli N. Is there an association between obstructive sleep apnea syndrome and periodontal inflammation? *Clin Oral Investig* (2016) 20:659–668. doi: 10.1007/s00784-015-1544-y

17. Latorre C, Escobar F, Velosa J, Rubiano D, Hidalgo-Martinez P, Otero L. Association between obstructive sleep apnea and comorbidities with periodontal disease in adults. *J Indian Soc Periodontol* (2018) 22:215–220. doi: 10.4103/jisp.jisp_38_18

18. Ahmad NE, Sanders AE, Sheats R, Brame JL, Essick GK. Obstructive sleep apnea in association with periodontitis: a case-control study. *J Dent Hyg* (2013) 87:188–199.

19. Sanders AE, Essick GK, Beck JD, Cai J, Beaver S, Finlayson TL, Zee PC, Loredo JS, Ramos AR, Singer RH, et al. Periodontitis and Sleep Disordered Breathing in the Hispanic Community Health Study/Study of Latinos. *Sleep* (2015) 38:1195–1203. doi: 10.5665/sleep.4890

20. Sales-Peres SHC, Groppo FC, Rojas LV, de C Sales-Peres M, Sales-Peres A. Periodontal Status in Morbidly Obese Patients With and Without Obstructive Sleep Apnea Syndrome Risk: A Cross-Sectional Study. *J Periodontol* (2016) 87:772–782. doi: 10.1902/jop.2016.150587

21. Gunaratnam K, Taylor B, Curtis B, Cistulli P. Obstructive sleep apnoea and periodontitis: a novel association? *Sleep Breath* (2009) 13:233–239. doi: 10.1007/s11325-008-0244-0

22. Chen Y, Metz JE, Gao H, Gao X. Association between obstructive sleep apnea and periodontitis in Chinese male adults: A cross-sectional study. *J Prosthet Dent* (2023) 130:80–86. doi: 10.1016/j.prosdent.2021.11.001

23. Nizam N, Basoglu OK, Tasbakan MS, Holthöfer A, Tervahartiala T, Sorsa T, Buduneli N. Do salivary and serum collagenases have a role in an association between obstructive sleep apnea syndrome and periodontal disease? A preliminary case-control study. *Arch Oral Biol* (2015) 60:134–143. doi: 10.1016/j.archoralbio.2014.09.006

24. Pico-Orozco J, Silvestre F-J, Carrasco-Llatas M, Silvestre-Rangil J. Influence of body mass index and obesity upon the association between periodontitis and sleep apnea-hypopnea syndrome. *Clin Oral Investig* (2021) 25:3929–3935. doi: 10.1007/s00784-020-03722-6

25. Nizam N, Basoglu OK, Tasbakan MS, Nalbantsoy A, Buduneli N. Salivary cytokines and the association between obstructive sleep apnea syndrome and periodontal disease. *J Periodontol* (2014) 85:e251-258. doi: 10.1902/jop.2014.130579

26. Al Habashneh R, Khassawneh B, Khader YS, Abu-Jamous Y, Kowolik MJ. Association Between Obstructive Sleep Apnea and Periodontitis Among Male Adults Attending a Primary Healthcare Facility in Jordan. *Oral Health Prev Dent* (2016) 14:157–164. doi: 10.3290/j.ohpd.a35002

27. Tamasas B, Nelson T, Chen M. Oral Health and Oral Health-Related Quality of Life in Children With Obstructive Sleep Apnea. *J Clin Sleep Med* (2019) 15:445–452. doi: 10.5664/jcsm.7672

28. Mukherjee S, Galgali SR. Obstructive sleep apnea and periodontitis: A cross-sectional study. *Indian J Dent Res* (2021) 32:44–50. doi: 10.4103/ijdr.IJDR_534_19

29. Chen Y, Chen X, Huang X, Duan Y, Gao H, Gao X. Analysis of Salivary Microbiome and Its Association With Periodontitis in Patients With Obstructive Sleep Apnea. *Front Cell Infect Microbiol* (2021) 11:752475. doi: 10.3389/fcimb.2021.752475

30. Kale SS, Kakodkar P, Shetiya SH. Assessment of oral findings of dental patients who screen high and no risk for obstructive sleep apnea (OSA) reporting to a dental college - A cross sectional study. *Sleep Sci* (2018) 11:112–117. doi: 10.5935/1984-0063.20180021
